# Optimal Decomposition of Service Level Objectives into Policy Assertions

**DOI:** 10.1155/2015/465074

**Published:** 2015-12-29

**Authors:** Yousef Rastegari, Fereidoon Shams

**Affiliations:** Department of Computer Engineering and Science, Shahid Beheshti University, P.O. Box 1983969411, Velenjak, Tehran, Iran

## Abstract

WS-agreement specifies quality objectives that each partner is obligated to provide. To meet quality objectives, the corresponding partner should apply appropriate policy assertions to its web services and adjust their parameters accordingly. Transformation of WS-CDL to WSBPEL is addressed in some related works, but neither of them considers quality aspects of transformation nor run-time adaptation. Here, in conformance with web services standards, we propose an optimal decomposition method to make a set of WS-policy assertions. Assertions can be applied to WSBPEL elements and affect their run-time behaviors. The decomposition method achieves the best outcome for a performance indicator. It also guarantees the lowest adaptation overhead by reducing the number of service reselections. We considered securities settlement case study to prototype and evaluate the decomposition method. The results show an acceptable threshold between customer satisfaction—the targeted performance indicator in our case study—and adaptation overhead.

## 1. Introduction

**Figure 1:**
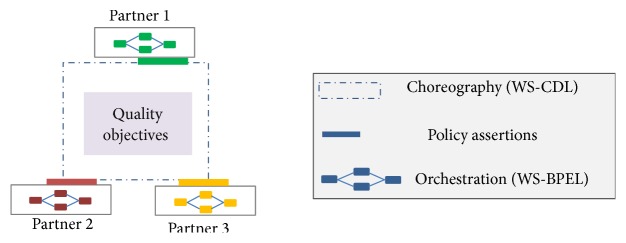
Policy assertions control WS-BPEL processes to satisfy quality objectives.

WS-CDL is the choreography standard to describe collaborative business processes. It shows a global view of all interactions among local WSBPEL processes. Since WS-CDL specifies only functional responsibilities of each partner, WS-agreement [1] is used to define service level objectives between a service provider and its consumers. It will assure consumers that they get the service they pay for and will obligate the service provider to achieve its service promises [2]. Therefore, service provider should explicitly manage quality properties of its local WSBPEL processes to realize service level objectives. Assessing the impact of new quality objectives on WSBPEL processes is not straightforward. Although quality objectives are defined at choreography level, they must be achieved at orchestration level by applying appropriate policy assertions (see Figure 1).

There are two types of transformation including model-driven (with the goal of integration) and formal (with the goal of verification) in the literature, but neither of them considers quality aspects of transformation nor run-time adaptation. The model-driven approaches translate a WS-CDL element to its respective replacement in terms of BPEL as well as WSCDL. This enables tracing down changes from choreography to orchestration and vice versa which is an important issue in the choreography adaptation scope. On the other hand, some studies formalize the WS-CDL elements. They tried to verify several aspects of service choreography like protocol compatibility, time constraints, and message ordering.

In this paper, we proposed a method to decompose service level objectives to WS-policy assertions. The assertions can be applied to WSBPEL elements and affect their run-time behaviors. While assertions describe what should be done with regard to quality objectives, adaptation strategies implement activities to achieve the objectives. For example, a performance assertion may cause the substitution of a slow service provider with a more efficient one (i.e., service reselection adaptation strategy).

The rest of the paper is organized as follows. The structure of WS-agreement is described in Section 2. In Section 3, we explain how to optimally decompose service level objectives into policy assertions and associate such policies with service subjects to which they should apply. To prototype and evaluate the proposed method, we define securities settlement case study in Section 4. This section is concluded by adaptation efficiency results.  Section 5 presents related works. Finally, Section 6 provides conclusions.

## 2. Structure of an Agreement

**Figure 2:**
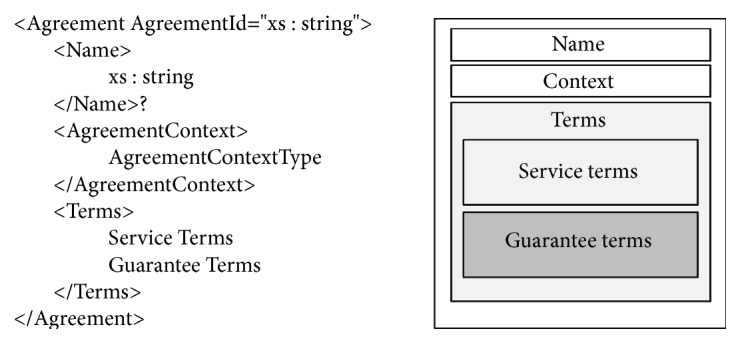
Structure of an agreement.

A service provider proposes an agreement template based on its capabilities, resources, and accepted agreement offers with other providers. As shown in Figure 2, an agreement is conceptually composed of several distinct parts. The section after the (optional) name is the context, which contains the meta-data for the entire agreement. It names the participants in the agreement and the agreement's lifetime. The next section contains the terms that describe the agreement itself.

An agreement defines service level attributes and service level objectives (SLOs). SLOs are expressed as assertions over service attributes and/or external factors such as date and time. Service terms contain* ServiceProperties* and* ServiceReference* to describe all aspects of service attributes. Guarantee terms contain* SeviceScope*,* QualifyingCondition*, and* ServiceLevelObjective* to specify a conditional assertion over a specific service term.

## 3. Proposed Optimal Decomposition Method

**Figure 3:**
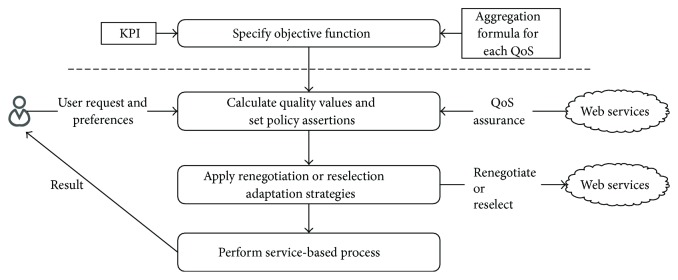
The process of adapting service-based process to user's preferences.

The process of handling user request is shown in Figure 3. For each user request, we identify appropriate quality values based on user's preferences and web services' quality assurance. To apply the identified quality values, we define their corresponding policy assertions. Then, to achieve the assertions, we realize renegotiation and reselection adaptation strategies which change the existing agreements or modify the existing service providers, respectively.

**Figure 4:**
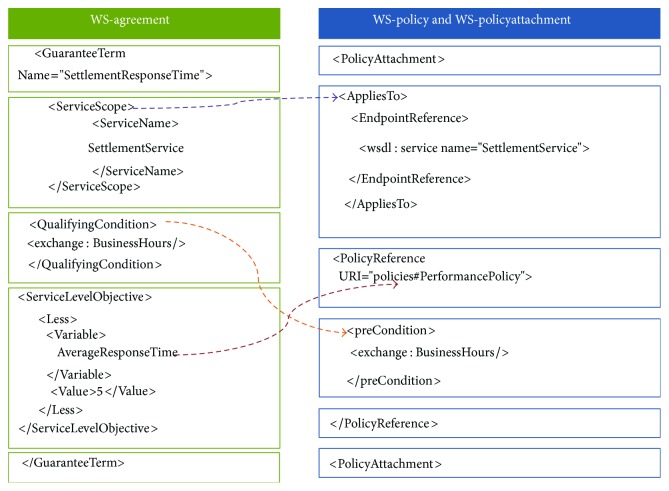
Integrating WS-agreement with orchestration-level standards.

To decompose (choreography-level) service level objectives into (orchestration-level) policy assertions, first we integrate their related standards (see Figure 4) and then present the decomposition method.

### 3.1. Policy Definition and Assignment

A policy contains one or more assertions; each one identifies requirements or capabilities of a policy subject. Policy assertions indicate low-level constraints on quality of services. For example, WS-ReliableMessaging [3] describes a protocol for reliable delivery of SOAP messages, or WS-Security describes enhancements to SOAP messaging to provide quality of protection [4].

As shown in Algorithm 1, we specified a generic format for defining domain-specific assertions. The policy* @id* attribute identifies the policy expression within the enclosing XML document. The* [QoS]Token* identifies what quality of service this assertion relates to (e.g., PerformanceToken). The* weight* attribute defines the impact of this service on the whole WSBPEL process; it mostly relates to user's preferences. The definition of quality of services and their measurement metrics are domain-specific; therefore, assertions are also expected to contain context of use. The* TokenType* is used to specify suitable metric for current context. For example, performance can be measured by average response time or throughput in Website Load or Job Submission System, respectively. The* TokenValue* indicates the value of a quality attribute. In our work, the assertions and their attributes and values are specified after decomposition of service level objectives.

Policies can be referenced internally or externally by* PolicyReference* element. The* PolicyReference @URI* attribute references a policy. In Algorithm 2, a policy with* #PerformancePolicy* identity is referenced externally by* wsdl:service* element. In this example, the performance policy will be applied to behaviors or aspects of the* SettlementService* as a whole.

WS-agreement supports both policy definition and policy assignment. WS-Policy is also a standard framework to model and express assertions, but it does not support policy assignment. Therefore, we used WS-PolicyAttachment as an extension for applying policies to service elements. WS-PolicyAttachment defines two general-purpose mechanisms for associating policies with service subjects to which they apply [16]. The policies can be applied to any part of a service such as endpoint, binding, port, portType, operation, and message. As shown in Algorithm 3, the external mechanism consists of* AppliesTo* and* PolicyReference* elements for identifying which service subjects are under control of which policies. The embedded mechanism adds* PolicyReference* besides a service subject to explicitly determine the scope of control.

### 3.2. Integration

Figure 4 shows the mapping between WS-agreement and “WS-policy and WS-PolicyAttachment” standards. Using policy attachment mechanisms, WS-agreement's* ServiceScope* is realized either by* AppliesTo* element or by adding* PolicyReference* to a specific service subject.

WS-agreement's* QualifyingCondition* is an optional condition that must be met (when specified) for a guarantee to be enforced. It is used to express a precondition for several service level objectives. Therefore, we add* preCondition* to* PolicyReference* to specify under what condition the policy should be applied. The type of* preCondition* is* xs:anyType*. It can be extended with assertion languages to address the requirements of the particular collaborative domain.

### 3.3. Decomposition

Performance requirements on business processes are specified as performance indicators with target values, which are to be achieved in a certain analysis period [17]. A key performance indicator (KPI) is a key metric which is measured by run-time monitoring mechanisms to prevent violation. It has both quality aspect (e.g., subprocess duration, service availability, and service security) and process aspect (e.g., number of ordered products and type of customer) [18]. In some cases, KPI measurement is not absolute; it relates to user's preferences. For example, customer satisfaction is a relative KPI; it relates to quality levels that fulfill user's preferences. Preference provides a mean by which a user can specify service quality levels that he would be satisfied with. For example, when an individual requests low cost and high performance process execution, all partners should consider these constraints while providing a service. While KPI forces quality requirements on a collaborative business process in general, SLOs specify constraints only on those service properties which are offered to service consumers. Therefore, service providers have to control service policies and apply adaptation strategies to comply with both collaborative agreement and user's preferences.

A business process includes several service-centric tasks; each one binds to a web service at run time. The web services communicate using different message exchange (composition) patterns such as sequence, parallel, loop, fork, and join. Here, we present a method to optimally decompose a service level objective and specify corresponding quality policies over service-centric tasks.

We applied nonlinear programming method to model our optimization problem. As shown below, the objective function is a specific KPI. KPI is defined based on four following factors: (1) context of use, (2) quality aggregation formula [19] per each quality of service, (3) service provider quality assurance, and (4) users' preferences. In (1), *Q*_*i*_ specifies *i*th quality of service, where *i* specifies number of effective qualities on KPI, *w*_*i*_ specifies the weight (importance) of *Q*_*i*_, and *F*_*Q*_*i*__ is the aggregation formula for *Q*_*i*_. *F*_*Q*_*i*__ would be different per each quality of service and composition pattern. For example, suppose a sequence of web services; the cost and availability aggregation formulas are specified as follows: *F*_cost_ = ∑_*i*=1_ *C*(*i*); *F*_availability_ = ∏_*i*=1_ *A*(*i*). The model of optimization problem is specified as follows.

Find the maximum (minimum) value of the objective function:

(1) $$\begin{array}{c} KPI=\underset{i=1}{\sum }w_{i}F_{Q_{i}}, \end{array}$$

where

(2) $$\begin{array}{c} F_{Q_{i}}=QoS\text{ aggregation formula for }Q_{i}, \end{array}$$

which is subject to the following:

(i) For each *Q*_*i*_, specify quality assurance of each service.
(ii) For each *Q*_*i*_, specify users' preferences.

## 4. Evaluation

### 4.1. Case Study: Securities Settlement [20, 21]

**Figure 5:**
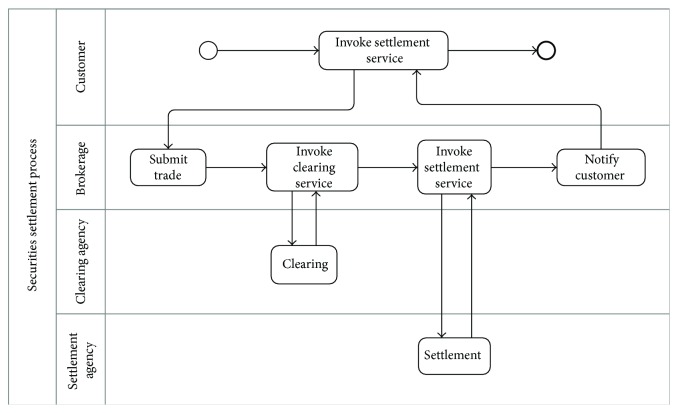
Securities settlement process.

Clearing and settlement are fundamental processes in financial markets. After the trade is executed, the record is submitted to the clearing agency, which matches the buyer and seller records and confirms that the counterparts agree to the terms. After the clearing process is performed, agencies fulfill the delivery requirements of the securities through settlement process. The settlement agency receives cash from buyers and securities from sellers and, at the end of the process, gives the securities to the buyer and the cash to the seller. The clients of the clearing and settlement agencies are brokerage companies. Brokerage companies can use different settlement and clearing agencies at the securities market. They receive trades from various customers like private individuals, institutional investors, or companies issuing or managing bonds. As shown in Figure 5, the customers can submit trades they made, to be settled by the brokerage companies through* settlement service* interface. From the brokerage company's perspective, it should bind to* clearing* and* settlement* web services to exchange securities. For the customers of the brokerage company, performance and cost of the* settlement service* are essential; they are the parameters of customer's preferences. The performance relates to the response time of both clearing and settlement processes. The cost relates to consumption of clearing and settlement web services and brokerage company's resources. The brokerage company can use different agencies at the securities market. After they receive a trade submission from a customer, they negotiate with existing agencies for new quality agreements or discover suitable agencies for satisfying the customer's preferences.

### 4.2. Prototype

As shown in Figure 5, the securities settlement process includes a sequence of two service-centric tasks (clearing and settlement). The objective function is customer satisfaction which depends on response time and cost quality attributes. The goal is to achieve the maximum customer satisfaction; therefore we should get the minimum response time and cost by considering both service quality assurance levels and user's preferences. As shown below, *Z* defines the aggregation formula for a sequence of two web services. We also consider 0.6 and 0.4 weight (importance) factors for response-time and cost, respectively. The maximum customer satisfaction is achieved when the value of *Z* is minimum. Response-time is abbreviated to rt; cost is abbreviated to *c*; clearing and settlement web services are indicated by their names, respectively:
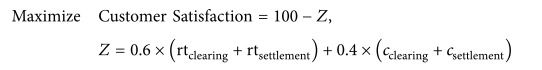
(3)

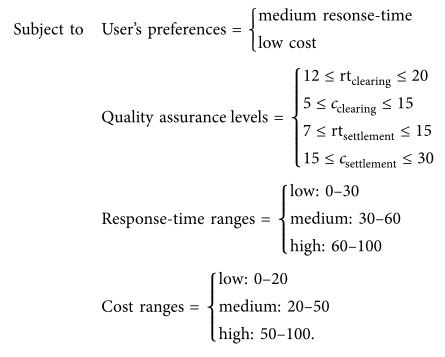
(4)Applying the simplex method [22] to this problem results in

(5) rtclearing=20,rtsettlement=10,cclearing=5,csettlement=15⟶Z=26.

So, the maximum value for customer satisfaction is

(6) $$\begin{array}{c} \text{Customer Satisfaction}=74. \end{array}$$

According to above results, the brokerage company should apply four new policies to service-centric tasks. The new policies are shown in Table 1. The brokerage company should also realize renegotiation adaption strategy to set new agreement offers based on new policies and send the offers to the clearing and settlement agencies.

### 4.3. Adaptation Efficiency

The decomposition method insists on staying with existing providers and negotiating for new quality agreements. But if the method does not find any solution to satisfy all constraints, the corresponding partner should apply other adaptation strategies such as reselection or reconfiguration. These strategies impose more overhead than renegotiation but may result in higher customer satisfaction, because they discover new service providers and set up an agreement which matches exactly what a user requested. This means that the decomposition method causes a bit of dissatisfaction but guarantees the lowest adaptation overhead. Thus, we set up an evaluation plan to measure the customer satisfaction and the adaptation overhead for the following scenarios, according to the securities settlement case study.

*Scenario  I*. Our proposed decomposition method is used. The method provides user's preferences by using existing clearing and settlement agencies. It insists on renegotiation. The reselection is used, only if the existing agencies cannot provide quality objectives.

The customer satisfaction was measured for 72 requests with different preferences. For each request, the preferences of response-time and cost were defined randomly as either low, medium, or high. These ranges were defined as follows: response-time {low: 5–40, medium: 41–60, high: 61–100}, cost {low: 5–40, medium: 41–70, high: 71–100}. At the beginning of the evaluation, the quality assurance levels of clearing and settlement web services were set as follows: clearing web service {response-time: 17–57, cost: 18–65}, settlement web service {response-time: 25–60, cost: 45–75}. We also considered 10 alternative candidates for clearing and settlement web services; each one offers different quality assurance levels.

The reselection adaptation strategy consists of the three following activities: (1) service discovery, (2) service selection, and (3) service binding. The negotiation for service level agreement is also done during service binding. Therefore, we considered (1) and (3) for the overhead value of realizing renegotiation and reselection adaptation strategies, respectively. The evaluation started based on the above configuration. The decomposition method was used to measure the customer satisfaction for each user's preferences, to see whether they can be accomplished by negotiating with existing web service providers (i.e., renegotiation) or selecting new ones (i.e., reselection).

*Scenario  II*. A reselection algorithm is used to discover and select new clearing and settlement agencies for each user's preferences.

Reselection algorithm finds new service providers that match exactly what user requested. As a consequence of selecting new suitable web services, the maximum customer satisfaction is achieved but it comes with more adaptation overhead.

As depicted in Table 2, the results show that the decomposition method (Scenario  I) achieves 87% customer satisfaction and 45% adaptation overhead reduction, in comparison with Scenario  II.

## 5. Related Work

There are some related works in transformation of WS-CDL to WSBPEL, but neither of them considers quality aspects of transformation nor run-time adaptation. In [5, 6], a model-driven transformation approach is proposed to drive BPEL process definitions from a global WS-CDL model. It proposes a mapping between WS-CDL and WSBPEL building blocks. In addition, the mapping can be used to generate WS-CDL description from existing WS-BPEL processes. In another model-driven approach, CDL2BPEL [7] algorithm translates WS-CDL to “BPEL and WSDL” elements, according to a knowledge base. The knowledge base contains generic patterns to translate a WS-CDL entity to its respective replacements in terms of BPEL as well as optional WSDL. The algorithm extracts WSDL interfaces from interactions and “tokens/token locators.” BPEL4Chor [8] is an intermediary language to align choreography and orchestration. BPEL4Chor adds a few constructs on top of BPEL to support all service interaction patterns. In addition to being an executable language, BPEL4Chor is an alternative for WS-CDL. More recently, a simple language (CDL) [9] was introduced to formalize the WS-CDL's participant roles and the collaborations among roles. They used SPIN model-checker to reason about properties that should be satisfied by the specified system automatically. Furthermore, in order to verify WS-CDL protocol mismatches, the transformation rules were proposed to describe the WS-CDL entities with timed automata [10] and colored Petri-net [11, 12] specifications. MOSES [13] is a QoS-based adaptation framework based on MAPE components. It is classified as an adaptive adaptation method. MOSES uses abstract composition to create new processes and also service selection to dynamically bind the processes to different concrete web services. MOSES is applicable where a service-oriented system is architected as a composite service. In [14], fuzzy controllers are applied to improve service-based applications based on context information (e.g., user, environment, and computational contexts). Beggas et al. [15] proposed middleware that calculates ideal QoS model using a fuzzy control system to fit context information and user preferences. Then, the middleware selects the best service among all variants having the nearest QoS value to the ideal. We used the following indicators to classify the related studies: what is the usage of approach? (integration, adaptation, and verification), which aspect does the approach cover? (functionality, quality), and what strategy is used to meet the research objective? The classification results are depicted in Table 3.

## 6. Conclusion

In this paper, we proposed a method to decompose WS-agreement service level objectives into WS-policy assertions. Assertions can be applied to web service elements and control their run-time behaviors. As a result, we can easily change quality objectives in a collaborative domain and track their effects on all relevant service parameters of each partner. In response to new assertions, service adaptation strategies (e.g., renegotiation, reselection) are applied to modify WSBPEL processes.

Considering renegotiation and reselection service adaptation strategies, the latter has more overhead; because a new service provider should be discovered, a new agreement offer should be sent to the provider and a new binding should be established. Apart from overhead of adaptation, there are other constraints like user's preferences and service quality assurance levels, which should be considered, if we want to get the best outcome for a business performance indicator. We modeled these constraints in an optimization problem using nonlinear programming method. We evaluated the method in securities settlement case study. The method reduces adaptation overhead and achieves acceptable customer satisfaction.

## Figures and Tables

**Algorithm 1:**
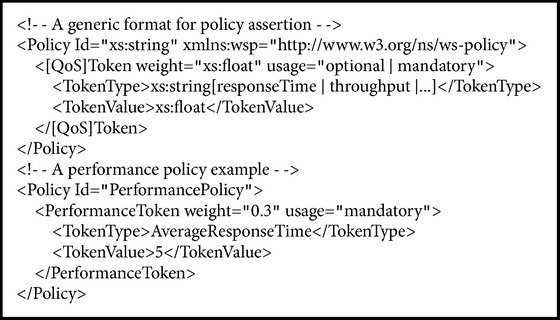
A generic format for service policy assertion.

**Algorithm 2:**
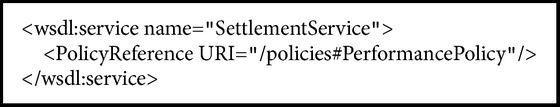
Policy reference is used to apply a performance policy to settlement service.

**Algorithm 3:**
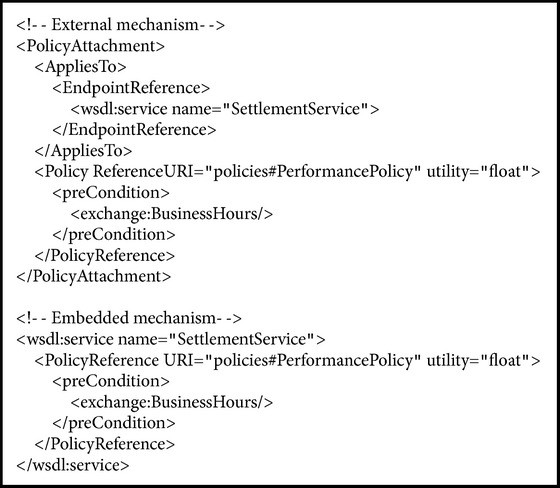
Policy attachment mechanisms.

**Table 1:** Performance and cost policies.

	Performance (it is measured by response-time)	Cost (it is measured by dollar)
Clearing service	<Policy Id="clearing-Performance">	<Policy Id="clearing-Cost">
	<PerformanceToken usage="mandatory">	<CostToken usage="mandatory">
	<TokenType>responseTime</TokenType>	<TokenType>dollar</TokenType>
	<TokenValue>20</TokenValue>	<TokenValue>5</TokenValue>
	</PerformanceToken>	</CostToken>
	</Policy>	</Policy>

Settlement service	<Policy Id="settlement-Performance">	<Policy Id="settlement-Cost">
	<PerformanceToken usage="mandatory">	<CostToken usage="mandatory">
	<TokenType>responseTime</TokenType>	<TokenType>dollar</TokenType>
	<TokenValue>10</TokenValue>	<TokenValue>15</TokenValue>
	</PerformanceToken>	</CostToken>
	</Policy>	</Policy>

**Table 2:** Adaptation efficiency results.

	Scenario I	Scenario II
Number of renegotiations	48	0
Renegotiation overhead	48	0
Number of reselections	24	72
Reselection overhead	72	216
Total adaptation overhead value (%)	120 (55%^*∗*^)	216 (100%)
Overhead reduction	45%	
Customer satisfaction value (%)	4591 (87%^*∗*^)	5221 (100%)
Customer dissatisfaction	13%	

^*∗*^The value is calculated as follows: (value of scenario I ÷ value of scenario II) × 100.

**Table 3:** Classification table.

	Usage	Aspect	Strategy
Our work	Integration, adaptation	Quality requirements, user's preferences	Model-driven approach to generate WS-policy assertions and tune BPEL processes based on user's preferences

[5, 6]	Integration	Functional requirements	Model-driven approach to drive BPEL processes from WS-CDL

CDL2BPEL [7]	Integration	Functional requirements	Knowledge-based approach to translate WS-CDL to “BPEL and WSDL” elements

BPEL4Chor [8]	Integration	Functional requirements	A new language which supports both BPEL and WS-CDL structures

CDL [9], timed automata [10], and colored Petri-net [11, 12]	Verification	Functional requirements	Formal specification and verification of WS-CDL

MOSES [13], fuzzy approaches [14, 15]	Adaptation	Quality, context information	By applying recomposition and reselection strategies to adapt an application to context information

## Symbols

### Parameters

*Q*_*i*_: *i*th quality of service
*w*_*i*_: Weight (importance) of *Q*_*i*_
*F*_*Q*_*i*__: Aggregation formula for *Q*_*i*_
rt: Response-time
*c*: Cost.

### Abbreviations

WS-CDL: Web service choreography description language
WSBPEL: Web service business process execution language
KPI: Key performance indicator
QoS: Quality of service.
